# Adapting across borders: Indonesian nurses' experiences in KSA

**DOI:** 10.1016/j.jtumed.2025.10.006

**Published:** 2025-11-20

**Authors:** Refina A. Resti, Ferry Efendi, Hakim Zulkarnain, Wedad M. Almutairi, Rifky O. Pradipta, I. Gede Juanamasta

**Affiliations:** a Faculty of Nursing, Universitas Airlangga, Surabaya, Indonesia; b School of Nursing and Midwifery, La Trobe University, Australia; c Research Center in Advancing Community Healthcare (REACH), Universitas Airlangga, Surabaya, Indonesia; d King Abdulaziz University, Jeddah, KSA; e Nursing Program, STIKes Wira Medika Bali, Denpasar, Indonesia

**Keywords:** Indonesian nurses, Life experience, Migration, KSA, الممرضات الإندونيسيات, تجربة الحياة, الهجرة, المملكة العربية السعودية

## Abstract

**Objective:**

The aim of this study was to explore the life experiences of Indonesian nurses living and working in KSA.

**Design:**

This qualitative study used a descriptive phenomenological qualitative design based on Colaizzi's method. The study included 20 Indonesian nurses who had lived and worked in KSA for a minimum of 1 year, and were recruited from hospitals. Data collection was conducted over a 1-month period in January 2024. Semi-structured interviews were conducted in appropriate settings and transcribed for analysis.

**Results:**

Four themes emerged: motivation for overseas employment; cross-cultural challenges; differences in nursing practices and professional standards; and benefits of living and working abroad. Among these themes, cross-cultural challenges, particularly in terms of communication, were perceived as complex issues by both patients and coworkers.

**Conclusion:**

The results of this study highlight the need for additional support for Indonesian nurses living in KSA, particularly regarding communication challenges. In addition, these findings have implications for workforce policies in both KSA and Indonesia, particularly regarding the preparation, support, and retention of migrant nurses.

## الملخص

**أهداف البحث:**

هدفت هذه الدراسة إلى استكشاف التجارب الحياتية للممرضات الإندونيسيات اللواتي يعشن ويعملن في المملكة العربية السعودية.

**طرق البحث:**

اعتمدت هذه الدراسة النوعية على تصميم وصفي نوعي قائم على منهج كوليزي. شملت الدراسة 20 ممرضة إندونيسية عشن وعملن في المملكة العربية السعودية لمدة لا تقل عن عام واحد، وتم اختيارهن من المستشفيات. تم جمع البيانات على مدى شهر واحد في يناير 2024. أُجريت مقابلات شبه منظمة في بيئات مناسبة، وتم نسخها لتحليلها.

**النتائج:**

برزت أربعة محاور رئيسية: الدافع للعمل في الخارج، والتحديات الثقافية؛ والاختلافات في ممارسات التمريض والمعايير المهنية؛ وفوائد العيش والعمل في الخارج. من بين هذه المحاور، اعتبر المرضى وزملاء العمل التحديات الثقافية، وخاصةً فيما يتعلق بالتواصل، قضايا معقدة.

**الاستنتاجات:**

تُبرز نتائج هذه الدراسة الحاجة إلى دعم إضافي للممرضات الإندونيسيات اللواتي يعشن في المملكة العربية السعودية، وخاصةً فيما يتعلق بتحديات التواصل. علاوةً على ذلك، لهذه النتائج آثارٌ على سياسات القوى العاملة في كلٍّ من المملكة العربية السعودية وإندونيسيا، لا سيما فيما يتعلق بإعداد ودعم واستبقاء الممرضات المهاجرات.

**Key points**

• This study highlights specific cross-cultural challenges faced by Indonesian nurses, such as dress codes, gender segregation, and communication barriers.
• This study provides a detailed comparison of nursing practices and professional standards between Indonesia and KSA, emphasizing differences in technology use, documentation systems, and licensing requirements.
• This study sheds light on the benefits of working in KSA, including financial stability, career development, and access to comprehensive employment benefits.

## Introduction

Nursing constitutes the most substantial share of the healthcare workforce, yet a growing nurse shortage has become a pressing global concern.^1^ Recent data have revealed that the nurse shortage remains a major obstacle in the global healthcare sector, by substantially affecting healthcare service standards, the global population's welfare, and the provision of comprehensive health coverage.^2^^,^^3^ Similarly, in KSA, the demand for nurses has continued to rise with population growth and demographic changes, and the number of available nursing staff remains insufficient.^4^^,^^5^

Recent statistics published by the World Bank in 2019 have indicated 5.7 Saudi nurses per 1000 individuals in the population, representing a 17.98 % increase from 2016 to 2019.^6^ In data from the World Health Organization published in 2017, KSA had the second-highest nurse-to-population ratio (with 55 nurses per 10,000 people) after the United Arab Emirates.^4^ On a global scale, the nurse-to-population ratio in KSA is lower than that in France (93 per 10,000), the United Kingdom (95 per 10,000), the USA (98 per 10,000), and Canada (105 per 10,000).^7^ By 2025, the demand for nurses in KSA was projected to double, thus creating a need for approximately 100,000 nursing positions to be filled by 2030.^8^^,^^9^ Nevertheless, Saudization has not yet effectively achieved its goals.^10^

Amid these challenges, globalization has created a more open and liberalized labor market^11^ that has enabled healthcare workers, including nurses, to seek job opportunities abroad more easily.^12^ With the increasingly connected global labor market, nurses have gained options to pursue careers in countries offering greater benefits and more attractive working conditions.^13^ In Indonesia, each year, more than 60,000 nurses graduate from 352 bachelor's degree programs and 474 diploma nursing programs, thus increasing the competition for available positions.^14^^,^^15^ Because of its excess of nursing graduates and limited domestic job opportunities, Indonesia has become a major source of professional nursing staff for countries facing nurse shortages.^16^ Although Indonesia is not the leading exporter of nurses, the country has gained recognition for its active participation in deploying nurses overseas.^17^ Therefore, Indonesia's international healthcare migration policy has prioritized broadening global employment opportunities for nurses.^18^ The destination countries for Indonesian migrant nurses are primarily in the Middle East: the top choices include KSA (26 %), Kuwait (18 %), the United Arab Emirates (3 %), and Qatar (2 %).^19^

KSA is the country with the highest percentage of Indonesian migrant nurses’ destinations, because it offers attractive employment packages including salaries, benefits, and professional growth opportunities.^20^^,^^21^ However, the transition for Indonesian nurses, as with other expatriates, requires substantial adjustments and poses challenges including cultural, social, and professional dimensions. These challenges can affect job satisfaction, mental health, and overall well-being (Nasser Saad Aldossari; Torke Aldosari; Faleh Aldosari, 2022;^22^). Because of cultural differences, Indonesian nurses in KSA encounter complex interactions, which are crucial to understanding their lived experiences. This phenomenon warrants further investigation. Previous studies have emphasized interacting factors driving nurses to seek employment overseas, including enhanced career opportunities, high earnings to provide for their families, and prospects for a supportive work environment and professional growth.^23^^,^^24^ However, no prior studies have investigated the experiences of Indonesian nurses living and working in KSA. Consequently, this research was aimed at providing new insights based on the perspectives of these nurses. The findings are expected to improve the advantages of healthcare worker migration for both nations while also offering meaningful insights for other countries.

## Materials and Methods

This study used a phenomenological qualitative method, which was selected to explore the experiences of nurses working in KSA. Descriptive qualitative methods, as outlined by Colaizzi, explore descriptive aspects to enhance understanding of the essence of daily lived experiences.

### Participants

The participants in this study were Indonesian nurses employed in KSA, who were recruited with the snowball sampling technique. This method was selected because of the scattered locations of the nurses and the researchers’ restricted access to the database of Indonesian nurses working in KSA. Moreover, snowball sampling in this study enhanced the diversity of included cities, ages, and employment spans.

Data collection was conducted over a 1-month period in January 2024. Potential participants were reached via phone and Facebook Messenger, and all interviews were conducted synchronously via Zoom. Although the data collection period was limited to 1 month, this timeframe was sufficient for several reasons. First, the focus of phenomenological research is depth rather than breadth; the aim is to obtain rich descriptions of lived experiences until data saturation is reached. Second, the snowball sampling strategy facilitated efficient recruitment by allowing participants to refer colleagues across diverse cities and hospitals, thereby expediting access to a varied sample. Third, interviews were scheduled flexibly, and participants' ability to choose their preferred times ensured that all 20 interviews could be conducted in a relatively short period without compromising quality. Therefore, although brief, the 1-month period was adequate to capture meaningful and saturated insights into Indonesian nurses’ experiences in KSA.

The inclusion criteria required candidates to be at least 20 years old, to have a minimum of 1 year of professional experience in KSA, and to be open to participating and sharing their personal and professional experiences. Interviews were scheduled after participants provided their consent. By the 20th interview, no new codes emerged. A review study has indicated that traditional qualitative research practices include a range of 5–35 participants.^25^ To support the analysis, a minimum of nine participants are needed to reach saturation.^26^

### Data collection

The study data were gathered via Zoom interviews, and participants selected their preferred times and locations to ensure easy and smooth interactions. Before the interviews, participants were briefed regarding the study, the voluntary nature of their involvement, and their right to withdraw from the study at any point. Written consent was obtained through WhatsApp. All interactions with participants were performed in their native language, Bahasa Indonesia.

The interviews were conducted through a semi-structured method. As a guide, the researchers developed the following set of questions: “Please can you tell me the reasons for you working as a nurse in KSA?” “What difficulties arise due to cultural differences between Indonesia and KSA?” “What challenges exist in communicating with patients or staff members you work with?” “What exams do you need to pass before or during your employment in KSA?” “What are the significant differences in nursing practices between working in Indonesia and KSA?” “What are the benefits or advantages you have gained while working and living in KSA?” The duration for data collection was approximately 60 min per participant. Verbal data were collected through digital audio recordings made during online interviews. The participants’ confidentiality was ensured by protection of the recordings with a password. The transcript data for each participant were subsequently analyzed and examined by the research team, with an emphasis on content, consistency, and thematic findings.

### Data analysis

Data transcription for this research was performed verbatim by two members of the research team. The transcribed data were subsequently analyzed in NVivo® software version 10, which aided in identifying and selecting statements considered relevant to the study. The thematic analysis followed Colaizzi's method. First, the interview transcripts were read multiple times to gain a comprehensive understanding and grasp their meaning. Second, the researchers identified significant phrases or statements. Third, relevant texts were extracted to develop concepts representing the main themes. Fourth, collaborative analysis sessions were held to resolve discrepancies and identify commonalities in the concepts. Fifth, initial themes and categories were formulated. Finally, preliminary findings were reviewed and discussed until all researchers reached a consensus regarding the final theme structure. The study's scientific trustworthiness was evaluated through the lens of the phenomenological cycle concepts.^27^ Key aspects such as credibility, dependability, confirmability, and applicability were examined to ensure the study's quality.^28^ Credibility was obtained by inviting participants to review and offer feedback on the initial findings. Interview transcripts, with emphasized statements and interpretations, were sent to the participants for clarification. Dependability was ensured by reviewing the analytical process and using a peer-review method for verification. Appropriateness was maintained through a documented diary of procedures and agreed concepts. Applicability was addressed by integrating data to help readers evaluate how the findings might be adapted to different contexts and applied by other researchers.

### Findings

A total of 20 participants were recruited for this study (participant characteristics in Table 1). Through analysis of the interview data, four themes emerged: motivation for overseas employment; cross-cultural challenges; differences in nursing practices and professional standards; and benefits of living and working abroad. A comprehensive summary of these themes reflecting the experiences of Indonesian nurses living and working in KSA is presented below.

**Table 1:** Sociodemographic characteristics of the study sample (n = 20).

Characteristics	n	%
**Age (years)**	24–47	
**Education**		
Nursing diploma	9	45
Bachelor's degree in nursing	8	40
Master's degree in nursing	3	15
**City**		
Ta'if	1	5
Makkah	11	55
Tabuk	1	5
Jizan	2	10
Riyadh	3	15
Madinah	1	5
Al Bahah	1	5
**Length of employment**		
<5 years	6	30
5–10 years	8	40
>10 years	6	30

### Theme 1: motivation for overseas employment

Many participants were driven to seek migrant work in KSA because of financial difficulties and expectations of a better future. Religious motivations were also substantial, because KSA was viewed as a place to fulfill spiritual practices such as Umrah and Hajj. Additionally, aspirations regarding education or professional experiences abroad influenced the decision. These factors were linked to the primary theme of the study: examining motivations for relocating to KSA to live and work.

1.1 Pursuing a Better Financial Life

Most participants pursued employment in KSA because of their families' financial struggles. They hoped that working there would improve their economic status and meet their family's needs.

“I chose to come here for one reason: the salary offered was higher compared to my previous workplace.” (P13)

“At that time, I thought that as someone from a village, I needed to change my fate and that of my family. Although I am a woman, I felt there was nothing wrong with going abroad.” (P15)

“In 2002, the only country offering opportunities to work abroad was KSA. Countries like Japan and Germany only started opening opportunities around 2010 and beyond. Therefore, my reason for choosing KSA was to improve my economic status.” (P4)

1.2 Religious Motivation

Some participants expressed gratitude and felt fortunate to work in KSA because it was a Muslim country, which enabled them to practice their religion without encountering difficulties. The participants revealed these sentiments, as shown in the following excerpts.

“The second reason that came to my mind at that time was that once we arrived in KSA, it would be easier for us to perform our religious practices.” (P3)

“Since KSA is a Muslim country, the people there strictly adhere to Islamic values. For example, when prayer time arrives, we must perform the prayers promptly, regardless of our personal wishes.” (P12)

The participants also emphasized additional benefits for foreign Muslims working in KSA. They described the increased opportunities to engage in Hajj or Umrah, the Islamic pilgrimages to Mecca. This spiritual incentive further motivated their migration to KSA.

“My primary motivation was actually to be able to perform Umrah or Hajj. Thank God, as a Muslim, I wanted to live in a country where the majority of the population is Muslim.” (P6)

“Why did I want to go to KSA? Because I wanted to visit the Nabawi Mosque frequently, perform Umrah more easily whenever I wished, and feel proud to work in the land of God.” (P17)

1.3 Career Advancement Goals

Migration to KSA for work was seen as an opportunity to improve well-being, particularly by increasing family income. Although financial reasons were the primary motivation, some participants also cited the achievement of personal goals and opportunities to gain more experience in the healthcare field as additional factors for working in KSA.

“... but my main reason was that I had just graduated in 2023, and I wanted my first work experience to help me learn and grow further.” (P18)

“Going to KSA wasn’t a definite choice, but while I was in college, I had a dream of becoming a professional nurse working in an international setting.” (P1)

“Actually, since I was young, my dream had been to travel abroad. I thought that working overseas would help me achieve that dream. Fortunately, I had family living here, which motivated me to come.” (P2)

### Theme 2: cross-cultural challenges

Participants faced many cross-cultural challenges in KSA, including strict dress codes, gender segregation, and substantial culture shock. They also struggled with the harsh climate, geographical differences, and communication barriers due to language and varying communication styles. This theme comprises three sub-themes.

2.1 Cultural Differences and Culture Shock

Participants shared their initial experiences in KSA, noting the blend of Islamic and Arabian cultural influences. They highlighted the importance of adapting to and respecting local customs, including dress codes and travel restrictions for women, and gained an understanding of the general demeanor of the population in both their professional and social interactions.

“Certainly, there are cultural differences, such as in food and the people themselves. The environment greatly influences our way of thinking.” (P6)

“We could not go out freely, could not walk with non-mahram (a close male relative whom a woman cannot marry under Islamic law), and could not easily greet people with smiles because it would be considered strange. The environment in KSA is also different from Indonesia; people there are not as fond of socializing, and Saudi society tends to be more closed-off.” (P11)

“... I experienced a kind of culture shock, particularly in terms of speaking and dressing habits. In Indonesia, we typically speak softly and politely, but here, people are accustomed to speaking loudly and with a high tone. This is considered normal here.” (P12)

2.2 Geographical Challenges

The geographical challenges faced by Indonesian nurses in KSA included extreme climate conditions, such as high temperatures and dry weather, which required substantial adaptation. These conditions markedly differed from Indonesia's tropical climate.

“Yes, indeed, some of the most surprising aspects when we arrived here were the extremely hot temperatures, reaching 45–48 °C. So, when we arrived, the difference was very noticeable. In Indonesia, the climate is tropical, sometimes cool, sometimes hot, but here, the heat is truly extreme.” (P13)

“I experienced a lot of culture shock, one of which was the fluctuating climate that required bodily adjustment.” (P14)

2.3 Communication Barriers

Most nurses acknowledged that effective communication was essential in their everyday nursing practice. The participants described communication with both patients and colleagues as particularly difficult.

“Communication between professionals and staff went smoothly because English was used.” (P18)

“We faced challenges in communicating with patients because not everyone in KSA speaks English. Our priority was to master English first; gradually learning Arabic here was also acceptable. Sometimes, we used sign language, and we were grateful that the patients could understand it.” (P17)

“Communication issues were quite challenging, even though I had received training from the company in Indonesia before being deployed to KSA. However, due to the limited time available after arriving here, I still felt that my communication skills were not fully developed.” (P5)

### Theme 3: differences in nursing practices and professional standards

Participants highlighted several differences in nursing practices and professional standards between KSA and Indonesia. These included the use of advanced medical equipment, integrated care systems, and the requirement to pass the Prometric examination for licensure. These variations substantially influenced their daily duties and professional growth.

3.1 Advanced Nursing Environment and Integrated Care

In KSA, participants worked with advanced medical equipment and technology, which improved the efficiency of nursing care. The healthcare system was also highly organized, with nurses assigned specific duties according to their specialization. This clear delineation of roles enhanced coordination and patient care.

“In KSA, we used various advanced medical devices compared to those in Indonesia, which made medical procedures faster. Additionally, all documentation was done electronically through the system, eliminating the need for paper.” (P18)

“The most striking difference is that in KSA, we truly work as professional nurses. In Indonesia, nurses often take on roles as medical assistants, such as cleaning wounds and handling instruments. Here, nurses and doctors work as a team, and nurses have nursing assistants to support their tasks.” (P1)

“The difference lies in the clear authority assigned to each profession, including nurses. Here, each profession, such as doctors, radiologists, and laboratory specialists, has its own area of responsibility and is not mixed. Nevertheless, we continue to work closely together.” (P9)

3.2 Licensing and Professional Standards

To work in KSA, participants were required to pass the Prometric exam. This rigorous licensing process ensures that high professional standards are met, thus highlighting the differences in regulatory frameworks and the strong focus on ongoing professional development.

“In KSA, there is a specific exam required to obtain a license, known as the Prometric exam. This exam is conducted entirely in English.” (P19)

“Here, there is an international registration process that must be completed first. After successfully passing the international registration, the next step is to register in KSA. Once all registration stages are finished, participants must take an exam. This exam has a format similar to the competency tests in Indonesia, but the difference is that it is conducted in English.” (P3)

“The exam here is quite similar to the exam for obtaining the Registration Certificate (STR). In KSA, the process involves a competency exam known as the Prometric exam. If we do not pass this exam within three attempts, we usually will not receive the license and will be sent back.” (P11)

### Theme 4: benefits of living and working abroad

Participants enjoyed several benefits of living and working in KSA, such as valuable employment benefits and improved professional opportunities. These advantages contributed to a rewarding and beneficial experience abroad.

4.1 Comprehensive Employment Benefits

Indonesian nurses in KSA received extensive benefits, including free medical care, housing, transportation, return tickets for leave, meals, and comprehensive insurance. These greatly enhanced their quality of life and job satisfaction.

“The provided benefits include accommodation and transportation. Additionally, we receive 36–40 days of leave per year. All round-trip transportation costs during leave are covered by them.” (P16)

“In terms of work, many facilities are provided. Firstly, the salary is quite good. Additionally, I receive allowances for accommodation, transportation, and meals. Moreover, I live in a dormitory equipped with a bus service.” (P18)

“Here, many benefits are provided. For example, if we fall ill, we can receive free medical treatment and undergo surgeries without the hassle of dealing with various administrative tasks. Additionally, there is a pension plan for retirement. If we work in Madinah or Mekkah, we also have the opportunity to participate in Hajj. During the Hajj season, we are included in the Hajj overtime program and receive a 1-month Hajj leave.” (P20)

4.2 Professional Experience and Networking

Working in KSA offered participants valuable professional experience and opportunities to build a broad network. Exposure to advanced technology and a variety of cases enriched their skills and created new career opportunities.

“The benefits I received included valuable experiences both inside and outside of work. Additionally, my language skills improved automatically, both in English and Arabic.” (P18)

“... we were able to enhance our skills and gain experience.” (P7)

“The benefits included experiences such as learning a new culture and acquiring a new language.” (P8)

## Discussion

This research explored the experiences of migrant nurses in KSA, with an emphasis on their reasons for seeking employment overseas. The findings revealed that their primary motivation was to earn higher incomes and improve their quality of life, given the economic challenges and low wages in their home countries. These findings were consistent with previous research indicating that economic motives are the primary factors in labor migration.^29^ Participants from economically disadvantaged backgrounds saw working abroad as a path to financial stability. Religious factors also influenced their decision. KSA's status as a center for Islamic practices, such as Umrah and Hajj, makes it an attractive destination for Muslim nurses.^30^ The opportunity to practice their religion more freely was a major motivator for participants, several of whom expressed gratitude for the ease of engaging in religious activities. These findings aligned with research highlighting the important roles of religious and cultural factors in migration decisions.^31^^,^^32^ Additionally, the drive to develop skills and knowledge through international work experience supported the continuation of participants' nursing careers. Research has indicated that working as international nurses enables acquisition of new skills, particularly clinical skills, assessment, and teamwork, as well as broader perspectives and awareness.^18^^,^^33^

The findings of this study indicated that migrant nurses in KSA experienced substantial cultural shock, particularly female nurses who had to adapt to strict dress codes and gender segregation. Previous studies have similarly identified cultural shock as a major challenge for migrant workers,^29^^,^^34^ emphasizing that successful integration depends on understanding and adapting to local norms. Participants in this study also reported persistent communication challenges, particularly with non-English-speaking patients and families, and often relied on body language, which was insufficient for conveying complex medical or emotional information. These results echo prior research showing that although non-verbal strategies can serve as temporary solutions, they cannot replace the need for clear verbal communication.^35^^,^^36^

Our findings also align with, and extend, research on cultural adaptation among migrant nurses in the Gulf region. Almutairi et al.^10^ have underscored the centrality of cultural competence in KSA's multinational nursing workforce, and warned that inadequate preparation exacerbates stress and undermines integration. Similarly, Alsadaan et al.^4^ have identified systemic and cultural challenges in Saudi nursing, including reliance on expatriates and retention difficulties. The cultural and communication difficulties, as well as the professional role adjustments reported by our participants, resonate strongly with these regional observations and underscore the need for context-specific strategies to support migrant nurses.

The findings of this study revealed substantial differences in nursing practices and professional standards between KSA and Indonesia, which affected daily tasks and the professional development of migrant nurses. In KSA, nurses use advanced medical equipment and an integrated healthcare system, which enhances the efficiency and quality of care.^37^ Modern medical equipment expedites procedures and decreases reliance on manual documentation, thus increasing work efficiency.^38^ Additionally, Indonesian nurses in KSA are required to pass the Prometric exam in English as a prerequisite for obtaining a license. This process emphasizes high professional standards and strict competencies, thus supporting the quality of medical services.^39^ In this context, the study participants indicated striking differences in the structure of care and medical team collaboration between Indonesia and KSA. In KSA, a strong emphasis is placed on teamwork, with clearly defined roles and responsibilities for each profession, thus promoting efficiency and enhancing the quality of care.^40^^,^^41^ In Indonesia, physicians typically supervise while nurses manage daily care and extra tasks. In KSA, the emphasis on teamwork highlights the importance of equality and recognition in improving work effectiveness and professional satisfaction.^42^

According to participants’ interviews, the final theme in this research was the additional value of working in KSA, which encompassed various important advantages gained by nurses as migrant workers in the nation. The findings showed that Indonesian nurses in KSA enjoyed a range of benefits, such as free medical facilities, accommodation, transportation, return tickets for leave, meals, and comprehensive insurance. These benefits not only enhance quality of life but also improve overall job satisfaction.^20^ In addition to these benefits, nurses also gain valuable professional experience and opportunities to build an extensive network.^43^ Working in KSA allowed participants to explore advanced technology and handle various medical cases, thus enriching their skills and opening new career opportunities. These findings were consistent with those from previous research indicating that the practical benefits and professional development gained from working abroad contribute to a rewarding and beneficial experience for migrant workers.^32^, ^44^

We acknowledge several study limitations that should be considered in interpreting the findings. The use of a snowball sampling technique and the relatively small sample size might have limited the representativeness of the participants. In addition, the 1-month data collection period might have constrained the depth of exploration. The generalizability of the results is also limited, because the study focused specifically on Indonesian nurses working in KSA rather than on migrant nurses more broadly. Furthermore, potential biases might have arisen from the use of an online format (e.g., Zoom) and the reliance on participants’ own networks for recruitment. To ensure transparency while maintaining the value of the research, we note these limitations as boundaries of the study rather than fatal flaws.

## Conclusion

Indonesian nurses in KSA gained economic benefits, professional opportunities, and additional spiritual value, but they faced challenges in understanding the local culture and communicating in English and Arabic. These findings underscore the importance of comprehensive preparation and sustained support. For Indonesia, strengthening pre-departure programs with an emphasis on language training and cultural orientation would better equip nurses for the realities of working abroad. For KSA, structured orientation and mentorship initiatives within healthcare institutions could facilitate smoother integration, enhance intercultural communication, and promote job satisfaction. At the policy level, bilateral agreements between countries are crucial to ensure ethical recruitment, protect migrant nurses’ rights, and support sustainable workforce planning. Future studies on the well-being and retention of Indonesian nurses in KSA should be conducted to determine how to address these factors in a sustainable manner.

### Relevance to clinical practice

• The findings may inform policies that enhance the migration experience for Indonesian nurses, including improved cultural preparation and language training before deployment.
• Saudi Arabian healthcare institutions may use these insights to develop better support systems for migrant nurses, such as orientation programs and peer mentoring initiatives.
• Indonesian nursing education programs may benefit from integrating international nursing competencies into curricula to better prepare graduates for global careers.
• The study contributes to discussions on ethical and sustainable international nurse recruitment practices, to ensure that both sending and receiving countries benefit from migration.

## Ethical approval

The research was approved by the Research Centre and Community Services at Universitas Airlangga, Surabaya, Indonesia (ethical certificate number: 3072-KEPK). Written consent was secured from participants before the interviews occurred. Participants were assured that their information would remain confidential and would be used solely for research purposes.

## Authors contributions

Refina Ayu Resti (RAR), Ferry Efendi (FE), and Hakim Zulkarnain (HZ) made substantial contributions to the study conception and design, acquisition of data, and analysis and interpretation of data. Wedad Matar Almutairi (WMA), Rifky Octavia Pradipta (ROP), and I Gede Juanamasta (IGJ) were involved in drafting the manuscript or revising it critically for important intellectual content. RAR, FE, HZ, WMA, ROP, and IGJ gave final approval for the publication of the final version. Each author has participated sufficiently in the work to take public responsibility for appropriate portions of the content.

All authors have critically reviewed and approved the final draft and are responsible for the content and similarity index of the manuscript.

## Conflict of interest

The authors have no conflict of interest to declare.
